# Relationship Between Peer Contact and Quality of Life in Dementia Caregivers

**DOI:** 10.1093/geroni/igaf122.3189

**Published:** 2025-12-31

**Authors:** Hailey Chatterton, Aimee Mooney, Heather Franklin, Allison Lindauer

**Affiliations:** Oregon Health & Science University, Portland, Oregon, United States; Oregon Health & Science University, Portland, Oregon, United States; Oregon Health & Science University, Portland, Oregon, United States; Oregon Health & Science University, Portland, Oregon, United States

## Abstract

Recent reviews of multi-component dementia caregiving interventions have indicated gaps in mechanism-driven social connection facets. Furthermore, relationships between peer support and quality of life among caregivers are not fully understood. Using data from a family caregiver psychoeducational intervention, Tele-STELLA (Support via Technology: Living and Learning with Advancing Dementia, NIA R01AG067546), we analyzed relationships between peer contact and caregivers’ quality of life (QoL), and between perceived contact helpfulness and likelihood of future contact. A merged dataset combined QoL assessments with self-reported peer contact data, totaling 423 observations from 153 participants. During intervention (n = 151) and follow-up (n = 142), participants reported past-month peer contact and future contact intentions. Generalized linear models assessed associations between helpfulness ratings and peer contact. QoL changes were analyzed using descriptive statistics and Cohen’s d for peer contact groupwise comparisons. Higher helpfulness ratings at baseline were associated with greater peer contact at follow-up (OR = 1.68, p = 0.03) and stronger intentions for future contact (OR = 5.49, p = 0.02). Among participants with “Good or Excellent” baseline QoL, peer contact had a small negative effect on QoL (d = -0.10); those with “Poor or Fair” QoL showed a moderate positive effect (d = 0.43). These values suggest that while peer contact had minimal association with QoL change for participants starting with higher QoL, it was moderately beneficial for those with lower baseline QoL. Additional research is needed to develop mechanism-informed interventions that promote social connection and improve QoL for dementia caregivers.

